# Allele Frequency of *APAF*1 Mutation in Holstein Cattle in Brazil

**DOI:** 10.3389/fvets.2022.822224

**Published:** 2022-02-23

**Authors:** Lukas Garrido Albertino, Ana Luísa Holanda Albuquerque, Julia Franco Ferreira, João Pedro Marmol Oliveira, Alexandre Secorun Borges, Thais Helena Constantino Patelli, José Paes Oliveira-Filho

**Affiliations:** ^1^São Paulo State University (UNESP), School of Veterinary Medicine and Animal Science, Botucatu, Brazil; ^2^School of Veterinary Science, Universidade Estadual do Norte do Paraná (UENP), Bandeirantes, Brazil

**Keywords:** *APAF*1, Holstein, *ARMS*-PCR, mutation, Bos taurus

## Abstract

*APAF*1 is an autosomal recessive inherited mutation, associated with Holstein haplotype 1 (HH1) and characterized by a substitution of cytosine for a thymine (c.1741C>T) in chromosome 5. The mutation causes fetal and embryonic loss, between 60 and 200 days of gestation, and reduced conception rate. The *ARMS*-PCR is considered a simple and low-cost method to determine single nucleotide polymorphism (SNP) with no need for genetic sequencing of the animal genome. This study aimed to verify the allelic frequency of *APAF*1 mutation in Brazilian Holstein cattle. A total of 248 Holstein DNA samples (210 cows and 38 bulls) were analyzed, and synthetic genes were manufactured to validate the primers developed by the authors. All animals assessed in this study were classified as wild-type for *APAF*1 mutation. The primers and protocol developed for the *ARMS*-PCR technique work with 100% specificity and efficiency since the amplicon formations are as expected according to the genotypes. In conclusion, the mutation responsible for *APAF*1 was not detected in the Brazilian Holstein cattle population assessed in this prevalence study, although it is not possible to affirm that *APAF*1 does not occur in Brazilian Holstein animals. The tetra-primer *ARMS*-PCR protocol for *APAF*1 mutation that has been validated here may be a relatively simple and economical method to determine the animals' genotype.

## Introduction

The *APAF*1 (apoptotic protease activating factor-1) protein is an important molecule in the cell apoptosis process (1) and central nervous system development during embryogenesis (2). The mutation is an autosomal recessive inherited disease, associated with HH1 and characterized by a substitution of cytosine for a thymine (c.1741C>T) in chromosome 5 (3). In Holstein cattle, the mutation causes fetal and embryonic loss between 60 and 200 days of gestation, and reduced conception rate, and was estimated to cause more than 500,000 abortions and a loss of US$450 million to the dairy industry (3). This mutation was associated with a common ancestor and carrier, the bull Pawnee Farm Arlinda Chief and its descendants, i.e., Walkway Chief Mark and Millu Betty Invahoe Chief. The three bulls, with S-W-D Valiant, were important sires in the history of the Holstein cattle breed and used for the study and validation of *APAF*1 mutation (3).

The diagnosis is made by the polymerase chain reaction (PCR) test and genetic sequencing of the animal, besides that, other tests like single-stranded conformation PCR (4), allelic specific PCR (5), and amplification refractory mutation system (*ARMS*-PCR) (6) were also used. The *ARMS*-PCR is considered a simple and low-cost method to determine SNP with no need for genetic sequencing of the animal genome (7), and it has been recently validated to determine the HH1 in Holstein cattle (6).

Studies performed in the United States (6, 8), France (9), Russia (10–12), Japan (5), and Poland (4) demonstrated that the prevalence of the mutation varies from 1.48% to 65%. However, according to known literature, there are no studies about this mutation in Brazil. Therefore, this study aimed to verify the allele frequency of *APAF*1 mutation in Brazilian Holstein cattle.

## Materials and Methods

### Ethical Approval Statement

This study was approved on February 11, 2019 by the Institutional Animal Care and Use Committee (0008/2019—CEUA—UNESP) and samples were collected under a strict confidentiality agreement to ensure the anonymity of establishments, owners, and animals.

### Sample Size Calculation and Samples Collection

A prevalence of 32.5% was assumed, based on a previous study in the United States (8), where 65% of the animals were heterozygous and considered the presence of wild-type animals. Based on the population of Holstein cattle registered in the Brazilian Association of Holstein Cattle Breeders (ABCBRH) in the states of Paraná (22.784 animals) and São Paulo (5.399 animals) in 2018, and using a 5% margin of error, and 95% confidence interval to calculate the sample size. The sample size recommended was 234 animals (OpenEpi).

A total of 248 Holstein DNA samples (210 cows and 38 bulls) were used in this study. These samples were obtained from five farms from São Paulo (4 farms) and Paraná (1 farm) states, Brazil. The inclusion criteria for the study were cows should have lactated or gestated at least twice, bulls should be used for breeding programs, and the animals should be registered in the breed association. A questionnaire was applied to characterize the dairy farm, herd, animal, and reproductive and productive indexes.

### *ARMS*-PCR Technique

To validate the primers and protocol developed for the *ARMS*-PCR technique, synthetic genes (homozygous T/T and wild-type C/C genotypes) were manufactured using Invitrogen GenArt Gene Synthesis (Life Science Solutions—Thermo Fisher Scientific, São Paulo, Brazil) (Supplementary Data). The oligonucleotide fragments were inserted into 5 μg dried p-MARQ (AmpR) plasmid DNA. The products were eluted with 50 μL of nuclease-free water (final concentration of 100 ng/μL) and used as a DNA sample in the PCR. The heterozygous (C/T) genotype gene was assembled by mixing 1.5 μL of homozygous and 1.5 μL wild-type genotypes genes.

The DNA was purified from 232 hair bulbs and 16 semen samples and stored at −20°C. *ARMS*-PCR primers (Table 1) were designed using online tools (Primer 1—Search Frame and BLAST—Basic Local Alignment Search Tool). PCR (30 μL) contained 3 μL of template DNA, 0.11 μM of each outer primer, 0.08 μM of each inner primer, 15 μL of GoTaq Green PCR Master Mix (Promega, Madison, WI), and 0.4 μL of nuclease-free water. Besides, a non-template control reaction was performed to check for the possible presence of contamination in the PCR preparations. The amplification conditions were as follows: initial denaturation at 95°C for 10 min, followed by 35 cycles of 95°C for 45 s, 60.1°C for 1 min, 72°C for 45 s, and a final extension at 72°C for 10 min. Amplicons were analyzed *via* 1.5% agarose gel electrophoresis, purified, and subject to Sanger direct sequencing. The obtained sequences and the electropherograms were analyzed using Sequencher (Gene Code Corporation, Ann Arbor, MI).

**Table 1:** *ARMS*-PCR primer sets used in this study.

**Primer sets**	**Primer sequences**	**Product (bp^a^)**
F inner (C)	GTGAACTGGAAACTTCAGAGGTTTATCTGC	286 bp
R inner (T)	CTGCTTGGCCTGCAGCTTAGCGTA	198 bp
F outer	TGATCTTGGCTCTGGTTATGTTTCTAAGCA	430 bp
R outer	GGCAAGCACCTATTTCAATGGACTCTTT	

a *Base pairs*.

## Results

The average age of cows and bulls assessed in the present study was 5 (±1.5) and 3 (±1.5) years, respectively. Of the 38 bulls assessed, 33 bulls (87%) were descendants from Pawnee Farm Arlinda Chief and its sons, of which 23 bulls were descendants from Walkway Chief Mark and 10 from S-W-D Valiant (Supplementary Figure). According to the questionnaire applied, we observed that 46 cows (46/210 cows; 22%) had episodes of embryonic absorption, fetal death, and spontaneous abortions between the 60th and 200th day of gestation. The average loss from reproductive problems, according to the farmer's answers, was US$8,000,00/year.

**Figure 1:**
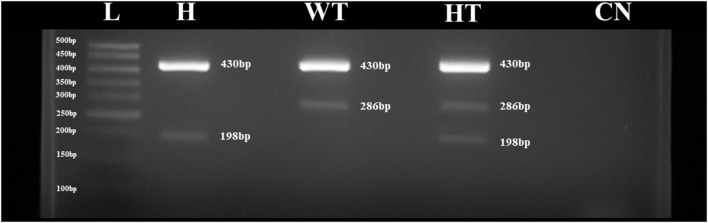
Agarose 1.5% gel with *ARMS*-PCR technique from *APAF*1 mutation using synthetic genes. (L) ladder 50 bp, (H) homozygous genotype (T), (WT) wild-type genotype (C), (HT) heterozygous genotype (C/T), and (CN) non-template control reaction.

**Figure 2:**
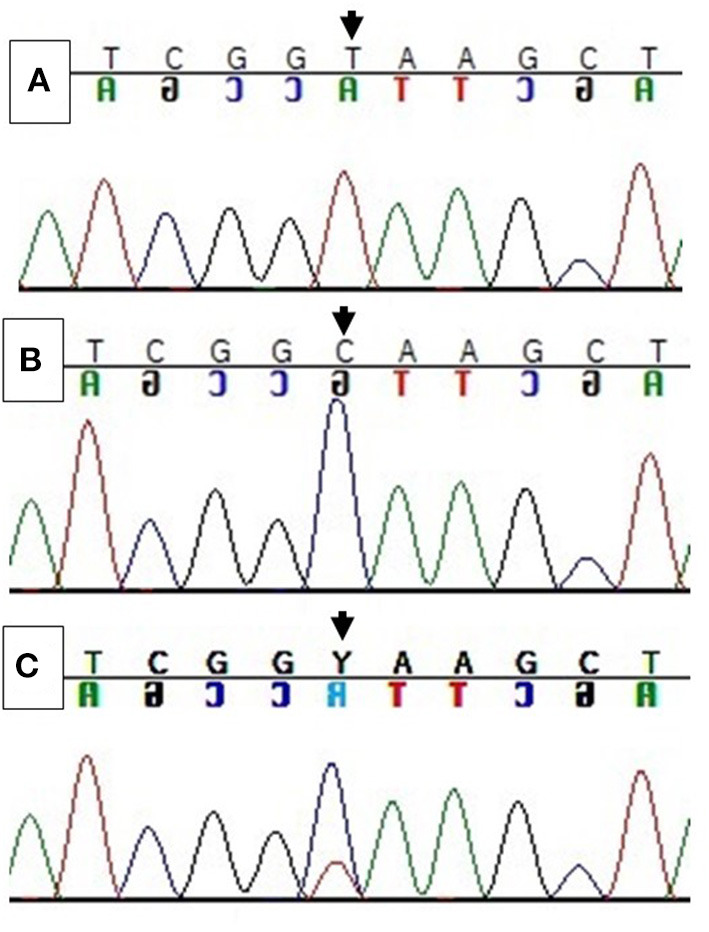
Partial electropherogram from *APAF*1 gene from **(A)** homozygous genotype (T), **(B)** wild-type genotype (C), and **(C)** heterozygous genotype (C/T). (Black arrow) point of mutation (c.1741C>T).

The *ARMS*-PCR primers and protocol developed here worked in the classification and determination of wild-type animals, forming amplicons according to their preview fragments [430 bp for outer primers and 286 bp for inner (C)]. The synthetic genes used as genotype controls worked well and formed amplicons of expected sizes, i.e., wild-type genotype gene formed a 430 bp for outer primer and 286 bp for inner primer (C), homozygous a 430 bp for outer primer and 198 bp for inner primer (T), and heterozygous a 430 bp for outer primer, 286 bp for inner primer (C), and 198 bp for inner primer (T) (Figure 1). The products were submitted to Sanger direct sequencing to confirm the specificity of the genes, especially the heterozygous genotype gene, assessed by mixing the other genes (Figure 2).

All animals assessed in this study were classified as wild-type for *APAF*1 mutation. That is, there were no carriers in this population. All samples were analyzed through the *ARMS*-PCR technique and submitted to Sanger direct sequencing with outer primer for confirmation of the genotype.

## Discussion

Although the genetic basis of the Holstein Brazilian herd has been formed by animals, semen, and embryos imported from Europe and North America (13) and previous studies in other countries have indicated the importance and impact of this mutation in the breed (3–5, 8–12), the *APAF*1 mutation has not been described in Brazil yet. In the present study, the first report that evaluated allele frequency of this mutation in Brazil, all animals assessed were homozygous for the wild-type allele, that is, no animal carried the mutant *APAF*1 allele. Chief's genetics are present in the Brazilian Holstein herd, in this study 33 bulls (33/38 bulls; 87%) evaluated were descendants of Chief and his sons, however, all these bulls were also homozygous for the wild-type allele. Seventy percent (23/33) of the bulls were descendants from Walkway Chief Mark and 30% (10/33) from S-W-D Valiant, important sires in the history of the Holstein breed and used for the study and validation of *APAF*1 mutation by Adams and collaborators (3). Considering that inbreeding is a widespread practice in bovine reproduction and production (3, 13), there are differences between the frequency of occurrence of *APAF*1 in some Holstein populations due to knowledge about HH1 and implementation of reproduction methods to eliminate the mutation of the breed (3).

No previous studies evaluated the dairy farm, herd, animal, and reproductive and productive indexes, only the prevalence of *APAF*1 mutation (3–5, 8–12). According to the questionnaire applied, we observed that 46 cows (46/210 cows; 22%) had episodes of embryonic absorption, fetal death, and spontaneous abortions between the 60th and 200th day of gestation. All cows were immunized against brucellosis, IBR, BVB, leptospirosis, mastitis, rabies, foot-and-mouth disease, keratoconjunctivitis, and *Clostridium* sp. Although these are the clinical signs caused by the mutation, none of them were associated with it, since all cows were wild-type, and we cannot affirm what caused these signs, since no diagnostic tests were performed.

The *ARMS*-PCR primers and protocol developed by the authors worked in the classification and determination of wild-type animals, forming amplicons according to expected fragments. Since no heterozygous (carriers) animals were found under the conditions in which this study was carried out, the authors opted to manufacture synthetic genes to validate the primers and protocol developed for the *ARMS*-PCR technique. The specificity of the synthetic genes was evaluated by Sanger direct sequencing, especially the heterozygous genotype gene, assessed by mixing the other genes, where, according to the electropherograms obtained, the genes customized had a 100% effective insert of the point of mutation.

The authors demonstrated that the primers and protocol developed for the *ARMS*-PCR technique for the *APAF*1 mutation work with 100% of specificity and efficiency since the amplicon formations are as expected according to the genotypes, even the determination of homozygous animals since this genotype cannot be observed in natural conditions (suffers from embryonic absorption, fetal death, and spontaneous abortions between the 60th and 200th day of gestation), differently from wild-type and heterozygous genotypes, as observed in other Holstein populations studies (3–5, 8–12).

In comparison with Kumar and collaborators' (6) protocol, our protocol has few differences, the sizes of amplicon fragments [189 bp for outer primers, 140 bp for inner (C), and 105 bp for inner (T) (6) vs. 430 bp for outer primers, 286 bp for inner (C), and 198 bp for inner (T)] and the amplification conditions [initial denaturation at 95°C for 3 min, followed by 35 cycles of 94°C for 30 s, 60° C for 30 s, 72°C for 1 min, and a final extension at 72°C for 10 min (6) vs. initial denaturation at 95°C for 10 min, followed by 35 cycles of 95°C for 45 s, 60.1° C for 1 min, 72°C for 45 s, and a final extension at 72°C for 10 min].

The amplification conditions are very relavent, where, according to Medrano and Oliveira (7), the melting temperature is considered the most important factor to achieve allele-specific amplification, and it can be optimized according to the region (high or low CG percentage) where the mutation is located.

In conclusion, the mutation responsible for *APAF*1 was not detected in this prevalence study in the Brazilian Holstein cattle population, although it is not possible to affirm that *APAF*1 does not occur in Brazilian Holstein animals, this disease seems to be extremely rare in the population. The primers and protocol developed and described by the authors for the *ARMS*-PCR technique have proven to be 100% specific and effective, and a relatively simple and economical method to determine the animals' genotype.

## Data Availability Statement

The datasets presented in this study can be found in online repositories. The names of the repository/repositories and accession number(s) can be found in the article/Supplementary Materials.

## Ethics Statement

The animal study was reviewed and approved by Animal Care and Use Committee from School of Veterinary Medicine and Animal Science.

## Author Contributions

LA and JO-F contributed to the conception and design of the study. LA, AA, JF, JO, and TP contributed to the samples collection. LA contributed to the processing of the samples. LA and AB contributed to the synthetic genes' manufactory. LA, TP, AB, and JO-F contributed to writing the first drafts of the manuscript. JO-F contributed to directing the project. All authors contributed to manuscript revision and read and approved the submitted version.

## Funding

This research was funded by Coordenação de Aperfeiçoamento de Pessoas de Nível Superior (CAPES)—Brasil—Finance Code 001.

## Conflict of Interest

The authors declare that the research was conducted in the absence of any commercial or financial relationships that could be construed as a potential conflict of interest.

## Publisher's Note

All claims expressed in this article are solely those of the authors and do not necessarily represent those of their affiliated organizations, or those of the publisher, the editors and the reviewers. Any product that may be evaluated in this article, or claim that may be made by its manufacturer, is not guaranteed or endorsed by the publisher.
