# Supplementary figures and images for: Allele Frequency of APAF1 Mutation in Holstein Cattle in Brazil
Source: Front Vet Sci. 2022 Feb 23;9:822224. doi: 10.3389/fvets.2022.822224 (PMC8904897; doi:10.3389/fvets.2022.822224)

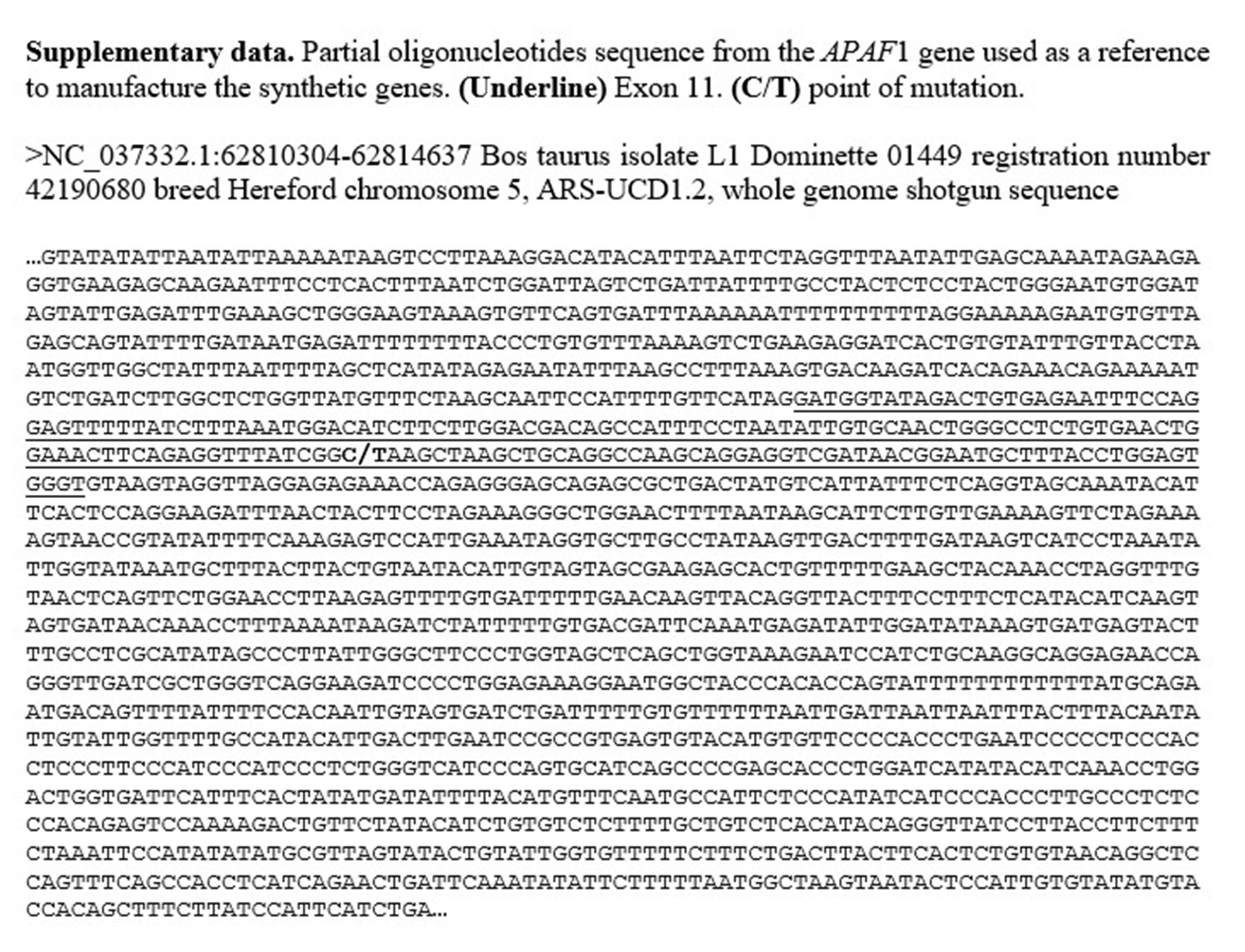

Supplement: Supplementary file 1 [file Image_1.JPEG]

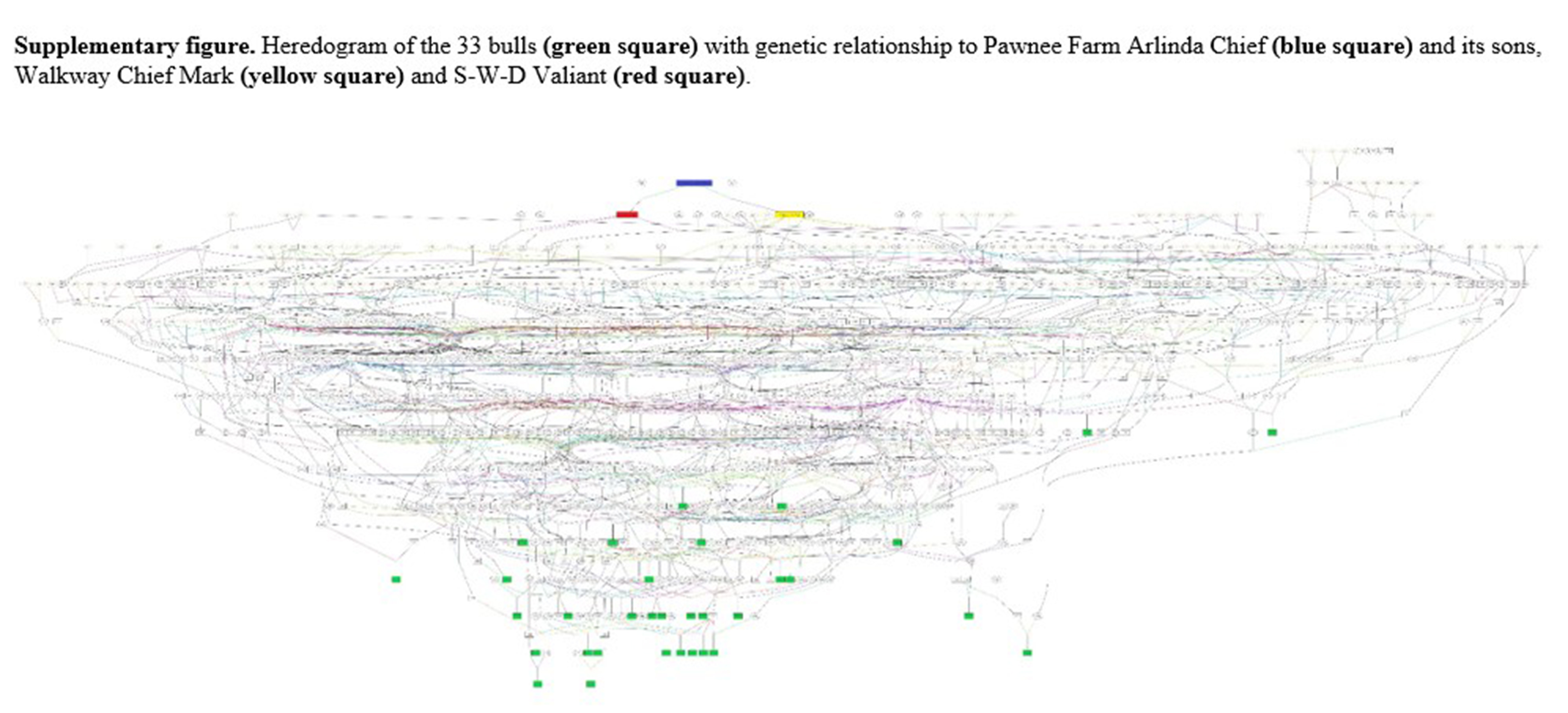

Supplement: Supplementary file 2 [file Image_2.jpg]
